# Comorbidity Between DSM–IV Alcohol and Drug Use Disorders: Results From the National Longitudinal Alcohol Epidemiologic Survey

**Published:** 1996

**Authors:** Bridget F. Grant, Roger P. Pickering

**Affiliations:** Bridget F. Grant, Ph.D., Ph.D., is the chief of the Biometry Branch, Division of Biometry and Epidemiology, National Institute on Alcohol Abuse and Alcoholism (NIAAA), Bethesda, Maryland. Roger P. Pickering, M.S., is a computer specialist in the Biometry Branch, Division of Biometry and Epidemiology, NIAAA

**Keywords:** comorbidity, AODD (alcohol and other drug use disorders), survey, epidemiology, United States, longitudinal study, diagnostic criteria

## Abstract

Research has not yet determined the answers to many questions regarding the comorbidity of alcohol and drug use disorders. Past studies often have not distinguished abuse from dependence and use and have not made diagnoses according to psychiatric classifications. This study relies on data from the 1992 National Longitudinal Alcohol Epidemiologic Survey, which attempts to address these concerns. The study demonstrates a pervasive co-occurrence of alcohol and drug use disorders in the general population. Further, the comorbidity of alcohol and drug dependence is found to be significantly greater than the comorbidity of alcohol and drug abuse.

Although much has been learned since the early 1970’s about the use of alcohol with other drugs, gaps remain in our understanding of the comorbidity of alcohol and drug use disorders (i.e., abuse and dependence). Our lack of knowledge concerning the relationship between alcohol and drug use disorders can be attributed to several factors. First, most studies have failed to differentiate alcohol and drug use from either abuse or dependence and, in fact, have used all three terms interchangeably. Second, few studies have provided for the requisite assessment of alcohol and drug use diagnoses according to current psychiatric classifications. Of these studies, the majority have been conducted among clinical or treated samples that are not well suited to the study of comorbidity (Rounsaville et al. 1982; Weiss et al. 1988). People in treatment are more likely to have multiple disorders than are people in the general population, thereby spuriously inflating estimates of the prevalence of comorbidity, a phenomenon referred to as Berkson’s bias (Berkson 1946).

Despite the bias inherent in the study of comorbidity in clinical samples, large general population surveys of the distribution of psychiatric comorbidity are rare. To date, only one general population survey has produced results bearing specifically on the co-occurrence of alcohol and drug use disorders (Helzer and Pryzbeck 1988). In this survey, entitled the Epidemiologic Catchment Area (ECA) study, 18,571 respondents were interviewed during the early 1980’s in a series of five community-based epidemiologic studies (Regier et al. 1984).

The ECA, however, has raised several historical and methodological issues that support further investigation of comorbid alcohol and drug use disorders. First, the study was conducted in the early 1980’s, and changes in the epidemiology of alcohol and drug use disorders and their associated comorbidity have rendered the ECA results outdated. In addition, the ECA utilized diagnostic criteria for alcohol and drug use disorders from the *Diagnostic and Statistical Manual of Mental Disorders, Third Edition* (DSM–III) (American Psychiatric Association 1980), a psychiatric classification that is no longer used in the field. Furthermore, as highlighted by Helzer and Pryzbeck (1988), the ECA was not a nationally representative sample of the U.S. population, and the measurement of alcohol and drug use disorders was not adequate. Alcohol and drug use disorders, as measured in the ECA, failed to account for the clustering requirement of the DSM–III. For example, in the ECA, a lifetime diagnosis of alcohol dependence was defined as the occurrence of a minimum of two alcohol symptoms on a lifetime basis. These symptoms, according to Helzer and Pryzbeck (1988), “may have been separated by a period of several years and there is no guarantee that there was ever a cluster of symptoms or alcohol problems occurring together.”

The purpose of the current study was to examine the comorbidity of alcohol and drug use disorders in a large representative sample of the United States population. In this study, alcohol and drug use disorders were classified according to the most recent psychiatric classification, the *Diagnostic and Statistical Manual of Mental Disorders, Fourth Edition* (DSM–IV) (American Psychiatric Association 1994), using a psychiatric assessment instrument that, importantly, defined alcohol and drug use disorders as syndromes, or the occurrence or clustering of symptoms within a period of time necessary to achieve a diagnosis.

## Methods

### Study Sample

This study is based on data from the 1992 National Longitudinal Alcohol Epidemiologic Survey (NLAES), which was designed and sponsored by the National Institute on Alcohol Abuse and Alcoholism, with fieldwork conducted by the Bureau of the Census. The NLAES consisted of direct face-to-face interviews with 42,862 adults, age 18 and older, who were randomly selected from a nationally representative sample of households. Interviews were conducted in the respondents’ homes, and proxies were not permitted. The study sample design of the NLAES included stratification and clustering^1^ as well as oversampling of blacks and young adults (ages 18 to 29) and is more fully described elsewhere (Grant et al. 1994). The household and sample person response rates for the NLAES were 91.1 percent and 97.4 percent, respectively.

### Diagnostic Assessment

Diagnoses of DSM–IV alcohol and drug use disorders were derived from the Alcohol Use Disorders and Associated Disabilities Interview Schedule (AUDADIS), a fully structured psychiatric interview designed to be administered by trained interviewers who are not clinicians (Grant and Hasin 1992). The AUDADIS includes an extensive list of symptom questions that operationalize the DSM–IV criteria for substance use disorders. Substance-specific diagnoses of abuse and dependence can be derived separately for alcohol, sedatives, tranquilizers, opioids (other than heroin), amphetamines, cocaine, cannabis (as well as tetrahydro-cannabinol [THC] and hashish), heroin, methadone, and hallucinogens. A prescription drug use disorder measure also was constructed to represent abuse of and/or dependence on sedatives, tranquilizers, opiates, and/or amphetamines. Similarly, any drug abuse and/or dependence measure was constructed to represent abuse and/or dependence on any drug excluding alcohol. Although the DSM–IV was not published until 1994, the specific diagnostic criteria of interest were known prior to conducting the NLAES (American Psychiatric Association 1991) and therefore were incorporated in their entirety within the AUDADIS.

Consistent with the DSM–IV, an AUDADIS diagnosis of past-year substance abuse required that a person exhibit a maladaptive pattern of substance use leading to clinically significant impairment or distress, as demonstrated by at least one of the following during the past year: (1) continued use despite a social or interpersonal problem caused or exacerbated by the effects of use, (2) recurrent use in situations in which substance use is physically hazardous, (3) recurrent use resulting in a failure to fulfill major role obligations, or (4) recurrent substance-related legal problems. An AUDADIS diagnosis of past-year substance dependence required that a person meet at least three of seven criteria defined for dependence during the past year, including the following: (1) tolerance; (2) withdrawal, or relief of or avoidance of withdrawal; (3) persistent desire or unsuccessful attempts to cut down or stop using; (4) spending much time obtaining a substance, using it, or recovering from its effects; (5) giving up or reducing occupational, social, or recreational activities in favor of substance use; (6) impaired control over use; and (7) continued use despite a physical or psychological problem caused or exacerbated by substance use.

Past-year diagnoses of alcohol and drug use disorders also satisfied the duration criteria of the DSM–IV. According to the DSM–IV, duration qualifiers associated with some, but not all, abuse and dependence criteria define the repetitiveness with which symptoms must occur in order to be counted as positive toward a diagnosis. Duration qualifiers are represented by the terms “recurrent,” “often,” and “persistent.” To satisfy a criterion associated with a duration qualifier, a respondent had to report having experienced at least one symptom two or more times during the past year or two or more symptoms of the criterion at least once during the same time period. To meet the criterion for withdrawal, which is defined as a syndrome or cluster of symptoms, two or more positive symptoms of withdrawal were required, each of which had to occur on at least two occasions. Corresponding prior to the past-year diagnoses of DSM–IV, alcohol and drug use disorders also were measured as syndromes, that is, the clustering of the required number of symptoms either (1) on most days for at least 1 month, (2) on and off for a few months or longer, or (3) at about the same time. Respondents classified with an alcohol- or drug-specific DSM–IV lifetime diagnosis encompassed all those who had ever experienced an episode of abuse and/or dependence either during the past year or prior to the past year.

### Statistical Analysis

Because of the complex survey design of the NLAES, variance estimation procedures that assume a simple random sample cannot be employed. To take into account the NLAES sample design, all standard errors of the prevalence estimates and comorbidity rates presented in this report were generated using Survey Data Analysis (SUDAAN) (Research Triangle Institute 1994), a software program that uses appropriate statistical techniques to adjust for sample design characteristics.

Associations between alcohol and drug use disorders were expressed in terms of odds ratios. Odds ratios and their 95-percent confidence intervals were derived from separate logistic regression analyses using the SUDAAN LOGISTIC program, which also adjusted for the complex sampling design of the NLAES. Because comorbidity is strongly influenced by important sociodemographic factors, two sets of odds ratios are presented: one set that has been adjusted for age, ethnicity, and sex and one set that consists of crude, or unadjusted, odds ratios. An odds ratio of greater than 1.0 reflects a positive association between the comorbid disorders and is statistically significant if its 95-percent confidence interval does not encompass the value of 1.0. In this report, comorbidity rates and odds ratios are not presented separately for opioids, heroin, or methadone because of their extremely low prevalence, and therefore imprecision, in the study sample.

The current analyses focused on what has been termed episode, or period, comorbidity, which is the co-occurrence of two or more psychiatric disorders during the same time period. Episode comorbidity should be contrasted with comorbidity viewed from the primary-secondary distinction, in which one of two or more comorbid disorders is designated as primary, usually on the basis of its onset at an earlier age. An important consequence of examining the co-occurrence of disorders from a period comorbidity rather than a primary-secondary perspective is that the odds ratios are equivalent regardless of whether an alcohol use disorder or a drug use disorder is designated as the index, or focal, disorder.

## Results

Table 1 shows the prevalence rates of lifetime DSM–IV alcohol and drug use disorders. The prevalence of lifetime alcohol use disorders was 18.2 percent, with 4.9 percent and 13.3 percent of the respondents classified as alcohol abusers and alcohol dependent, respectively. For all drugs combined, slightly more respondents were classified with abuse (3.1 percent) than dependence (2.9 percent). Prevalences of lifetime sedative, tranquilizer, and hallucinogen abuse and dependence combined were less that 1.0 percent. For the remainder of the drugs, lifetime abuse and/or dependence was approximately 1.5 percent for amphetamines and cocaine, 2.0 percent for prescription drugs, and 4.6 percent for cannabis. With the exception of cocaine and alcohol, the prevalences of abuse diagnoses equaled or exceeded the corresponding dependence diagnoses.

Table 2 shows the comorbidity rates between DSM–IV alcohol and drug use disorders. Among respondents with any drug use disorder, 69.4 percent were classified with an alcohol use disorder, compared with 14.9 percent of the respondents with no history of a drug use disorder. The prevalences of alcohol abuse and alcohol dependence combined among respondents with prescription drug, sedative, tranquilizer, amphetamine, cannabis, cocaine, and hallucinogen abuse or dependence combined were quite high, ranging from approximately 60.0 to 80.0 percent. In general, the prevalences of alcohol abuse among respondents with drug abuse (11.0 to 16.4 percent) were much lower than those associated with drug dependence (70 to 85 percent).

The results of table 2 were calculated under the assumption that respondents with drug use disorders represent the exposed group. One could easily designate, however, respondents with alcohol use disorders as the exposed group, as indicated in table 3, and then calculate the odds of a drug use disorder in that exposed group relative to the odds of a drug use disorder in the unexposed group or in those without an alcohol use disorder. Regardless of the specification of exposed and unexposed groups, the odds ratios calculated from the results presented in tables 2 and 3 will be identical.

As seen in table 3, the prevalence of any drug use disorder among respondents with a history of an alcohol use disorder was 23.1 percent, compared with 2.3 percent among respondents who had not had an alcohol use disorder. Respondents classified with an alcohol use disorder were at approximately 10 times the risk of having a drug use disorder as were those with no alcohol use disorder. The associations^2^ between hallucinogen (OR = 17.1) and tranquilizer (OR = 16.5) abuse and/or dependence and alcohol abuse and/or dependence were greater than those for sedatives (OR = 14.6), cocaine (OR = 12.0), amphetamines (OR = 11.4), cannabis (OR = 9.3), or overall prescription drug abuse and/or dependence (OR = 10.6). For all drug categories examined, the associations between drug abuse and alcohol abuse were much smaller than the corresponding dependence association. In all cases, respondents classified with alcohol abuse were at approximately two times the risk of having a drug use disorder as were those without a history of alcohol abuse.

## Discussion

Virtually all the odds ratios presented in this study were significantly greater than 1.0, demonstrating that the comorbidity of alcohol and drug use disorders is pervasive in the general population. Among those with a lifetime DSM–IV drug use disorder, 69.4 percent experienced an alcohol use disorder, a comorbidity rate significantly greater than the population base rate of alcohol use disorders (18.17 percent). Conversely, 23.1 percent of the respondents with an alcohol use disorder also reported a drug use disorder, a comorbidity rate significantly greater than the population base rate of drug abuse and dependence combined (6.1 percent). These results strongly support the growing trend in recent years toward integrating drug abuse and alcoholism treatment programs.

The findings from this study largely confirm the results of the ECA conducted in the early 1980’s. The associations between alcohol and drug abuse, dependence, and abuse and/or dependence were 2.9, 10.9, and 9.6, respectively. These odds ratios were strikingly similar to those reported by Helzer and Pryzbeck (1988) from the ECA (OR’s = 3.9, 11.2, and 7.2, respectively). Although not strictly comparable, the associations found in the study for abuse and/or dependence closely corresponded to those reported from the ECA for sedatives (NLAES: OR = 14.6; ECA: OR = 16.9), amphetamines (NLAES: OR = 11.4; ECA: OR = 11.1), and cannabis (NLAES: OR = 9.3; ECA: OR = 6.0). In contrast, the association between alcohol abuse and/or dependence and hallucinogen abuse and/or dependence was greater in the NLAES (OR = 17.1) than in the ECA (OR = 10.9), whereas the corresponding association for cocaine was greater in the ECA (OR = 36.3) than in the NLAES (OR = 12.0) (Regier et al. 1990). Discrepancies between the NLAES and ECA findings may result from differences in the sampling frame and sample size, the diagnostic interview schedules, or the diagnostic criteria used to formulate the diagnoses. It is also likely that changes in alcohol and drug use practices in the United States during the 10-year period since the ECA was conducted have altered the relationships between alcohol and drug use disorders.

The associations between alcohol abuse and drug abuse, regardless of drug type, were consistently found to be smaller than those between alcohol and drug dependence. These results provide some support for the widespread view that abuse is a less severe form of the disorder than dependence and that fewer complications, such as comorbid drug abuse, would be expected with a less severe disorder. Recall that the DSM–IV defines substance abuse separately from dependence, as social, occupational, legal, and interpersonal consequences arising from drinking. The manual relegates indicators of patterns of compulsive drinking (e.g., impaired control over drinking, giving up important activities to drink) and tolerance and withdrawal to the dependence category. Alternatively, the abuse associations may be smaller than the dependence associations because the dependence construct may be defined more diffusely than the abuse construct.

The results of this study highlight the importance of comorbid alcohol use disorders and drug use disorders. Alcohol use disorders have been documented to increase both morbidity and mortality among persons with drug use disorders, primarily due to liver dysfunction (Maddux and Elliott 1975), overdose deaths (Baden 1970), and continued drug use (Mezritz et al. 1975). In view of the extent of comorbid alcohol and drug use disorders in the general population and the resultant adverse consequences, it would seem imperative to sharpen our efforts with regard to identifying comorbid patients and designing specific treatment modalities for them. With reference to detection, health professionals in all treatment sectors should be alert to comorbid alcohol and drug use disorders, particularly professionals in primary care, in which physicians have been shown to be less successful in diagnosing patients with alcohol and drug use disorders (Gold and Dackis 1986).

Although the results of this study have answered basic questions about the distribution of comorbidity between alcohol and drug use disorders, future research using the NLAES data will focus on defining the heterogeneity of people with alcohol and drug use disorders. This research will compare respondents with comorbid alcohol and drug use disorders with respondents who have alcohol use disorders but no history of drug use disorders and respondents who have drug use disorders but no history of alcohol use disorders. This comparison would identify important subgroups of substance use disorders that may be distinguished by differences in sociodemographic factors, family history profiles, and associated psychopathology. The identification of important subgroups of substance use disorders should both further our efforts to more readily identify those with alcohol and drug use problems and aid in specific treatment planning.

## Figures and Tables

**Table 1:** Prevalence of Lifetime DSM–IV^1^ Alcohol and Drug Use Disorders: United States, 1992

Disorder	Prevalence (%)	S.E.^2^
Alcohol abuse and/or dependence	18.17	(0.27)
Alcohol abuse only	4.88	(0.13)
Alcohol dependence	13.29	(0.22)
Any drug abuse and/or dependence	6.05	(0.15)
Any drug abuse only	3.14	(0.11)
Any drug dependence	2.91	(0.10)
Prescription drug abuse and/or dependence	2.01	(0.08)
Prescription drug abuse only	0.98	(0.06)
Prescription drug dependence	1.03	(0.06)
Sedative abuse and/or dependence	0.64	(0.04)
Sedative abuse only	0.30	(0.03)
Sedative dependence	0.34	(0.03)
Tranquilizer abuse and/or dependence	0.63	(0.05)
Tranquilizer abuse only	0.31	(0.03)
Tranquilizer dependence	0.32	(0.03)
Amphetamine abuse and/or dependence	1.48	(0.07)
Amphetamine abuse only	0.76	(0.05)
Amphetamine dependence	0.72	(0.05)
Cannabis abuse and/or dependence	4.64	(0.14)
Cannabis abuse only	2.86	(0.11)
Cannabis dependence	1.78	(0.08)
Cocaine abuse and/or dependence	1.66	(0.07)
Cocaine abuse only	0.64	(0.04)
Cocaine dependence	1.02	(0.06)
Hallucinogen abuse and/or dependence	0.59	(0.04)
Hallucinogen abuse only	0.30	(0.03)
Hallucinogen dependence	0.29	(0.03)

1 DSM–IV = Diagnostic and Statistical Manual of Mental Disorders, Fourth Edition.

2 SE = standard error.

**Table 2:** Lifetime Prevalence of Selected DSM–IV^1^ Alcohol Use Disorders Among Respondents With and Without Corresponding DSM–IV^1^ Drug Use Disorder; United States, 1992.

Drug Use Disorder	Prevalence of Alcohol Use Disorders^2^ Among Respondents			

	With Corresponding Drug Use Disorder^3^		Without Corresponding Drug Use Disorder^3^	
	
	%	S.E.^4^	%	S.E.^4^
Any drug abuse and/or dependence	69.4	(1.04)	14.9	(0.25)
Any drug abuse only	16.4	(1.17)	4.5	(0.13)
Any drug dependence	65.3	(1.53)	11.7	(0.21)
Prescription drug abuse and/or dependence	73.4	(1.74)	17.0	(0.27)
Prescription drug abuse only	13.4	(2.27)	4.8	(0.13)
Prescription drug dependence	68.9	(2.50)	12.7	(0.22)
Sedative abuse and/or dependence	80.1	(2.57)	17.8	(0.27)
Sedative abuse only	13.0	(3.81)	4.9	(0.13)
Sedative dependence	80.6	(3.38)	13.1	(0.22)
Tranquilizer abuse and/or dependence	80.7	(3.61)	17.8	(0.27)
Tranquilizer abuse only	11.2	(3.52)	4.9	(0.13)
Tranquilizer dependence	79.3	(4.06)	13.1	(0.22)
Amphetamine abuse and/or dependence	75.9	(2.05)	17.3	(0.27)
Amphetamine abuse only	13.5	(2.67)	4.8	(0.13)
Amphetamine dependence	70.0	(3.07)	12.9	(0.22)
Cannabis abuse and/or dependence	71.0	(1.22)	15.6	(0.25)
Cannabis abuse only	16.6	(1.19)	4.5	(0.13)
Cannabis dependence	69.5	(2.00)	12.3	(0.21)
Cocaine abuse and/or dependence	76.4	(1.86)	17.2	(0.26)
Cocaine abuse only	15.3	(2.80)	4.8	(0.13)
Cocaine dependence	71.2	(2.42)	12.7	(0.22)
Hallucinogen abuse and/or dependence	85.4	(2.25)	17.8	(0.27)
Hallucinogen abuse only	11.0	(3.62)	4.9	(0.13)
Hallucinogen dependence	84.7	(3.43)	13.1	(0.22)

1 DSM–IV = Diagnostic and Statistical Manual of Mental Disorders, Fourth Edition.

2 Abuse and/or dependence, abuse only, or dependence only.

3 Comparisons between alcohol abuse versus drug-specific abuse, alcohol dependence versus drug-specific dependence, and alcohol abuse and/or dependence versus drug-specific abuse and/or dependence.

4 SE = standard error.

**Table 3:** Lifetime Prevalence and Odds Ratios of Selected DSM–IV^1^ Drug Use Disorders Among Respondents With and Without Corresponding DSM–IV^1^ Alcohol Use Disorder: United States, 1992

Drug Use Disorder	Prevalence of Drug Use Disorders^2^ Among Respondents				Odds Ratio	Adjusted Odds Ratio^5^	95% Confidence Interval

	With Corresponding Alcohol Use Disorder^3^		Without Corresponding Alcohol Use Disorder^3^				
	
	%	S.E.^4^	%	S.E.^4^			
Any drug abuse and/or dependence	23.1	(0.61)	2.3	(0.09)	13.0	9.6	(8.6, 10.6)
Any drug abuse only	10.6	(0.79)	2.8	(0.10)	4.2	2.9	(2.4, 3.5)
Any drug dependence	14.3	(0.55)	1.7	(0.70)	14.2	10.9	(9.5, 12.6)
Prescription drug abuse and/or dependence	8.1	(0.38)	0.7	(0.05)	13.4	10.6	(8.8, 12.9)
Prescription drug abuse only	2.7	(0.49)	0.9	(0.06)	3.1	2.2	(1.5, 3.2)
Prescription drug dependence	5.3	(0.34)	0.4	(0.04)	15.2	12.6	(9.9, 16.1)
Sedative abuse and/or dependence	2.8	(0.22)	0.2	(0.02)	18.6	14.6	(10.6, 20.2)
Sedative abuse only	0.8	(0.25)	0.3	(0.03)	2.9	2.1	(1.1, 4.1)
Sedative dependence	2.0	(0.22)	0.1	(0.01)	27.6	22.0	(14.5, 33.5)
Tranquilizer abuse and/or dependence	2.8	(0.23)	0.2	(0.02)	19.4	16.5	(11.5, 23.6)
Tranquilizer abuse only	0.7	(0.21)	0.3	(0.03)	2.5	1.8	(0.9, 3.7)
Tranquilizer dependence	1.9	(0.21)	0.1	(0.02)	25.5	22.2	(13.5, 36.5)
Amphetamine abuse and/or dependence	6.2	(0.33)	0.4	(0.04)	15.0	11.4	(9.1, 14.5)
Amphetamine abuse only	2.1	(0.45)	0.7	(0.05)	3.1	2.2	(1.4 3.4)
Amphetamine dependence	3.8	(0.30)	0.3	(0.03)	15.8	12.3	(9.1, 16.6)
Cannabis abuse and/or dependence	18.1	(0.55)	1.6	(0.08)	13.3	9.3	(8.2, 10.5)
Cannabis abuse only	9.7	(0.72)	2.5	(0.10)	4.2	2.9	(2.4, 3.5)
Cannabis dependence	9.3	(0.48)	0.6	(0.05)	16.3	11.7	(9.7, 14.2)
Cocaine abuse and/or dependence	7.0	(0.33)	0.5	(0.04)	15.6	12.0	(9.6, 15.0)
Cocaine abuse only	2.2	(0.54)	0.8	(0.07)	3.6	2.5	(1.6, 3.9)
Cocaine dependence	5.5	(0.34)	0.3	(0.03)	17.0	13.5	(10.6, 17.2)
Hallucinogen abuse and/or dependence	2.8	(0.22)	0.1	(0.01)	27.1	17.1	(12.0, 24.6)
Hallucinogen abuse only	0.7	(0.24)	0.3	(0.03)	2.4	1.6	(0.8, 3.2)
Hallucinogen dependence	1.8	(0.22)	0.1	(0.01)	36.8	23.2	(13.5, 40.0)

1 DSM–IV = Diagnostic and Statistical Manual of Mental Disorders, Fourth Edition.

2 Abuse and/or dependence, abuse only, or dependence only.

3 Comparisons between alcohol abuse versus drug-specific abuse, alcohol dependence versus drug-specific dependence, and alcohol abuse and/or dependence versus drug-specific abuse and/or dependence.

4 SE = standard error.

5 Odds ratio adjusted for age, sex, and ethnicity.

## Footnotes

1 For a definition of these and other technical terms, see glossary, p. 72.

2 Expressed in terms of odds ratios (OR) adjusted for age, sex, and ethnicity.

## Glossary

Cluster sampling: A sampling method in which each sampling unit is a collection of persons, units, or elements of interest.
Confidence interval for an odds ratio: When 95-percent confidence intervals are constructed around a sample estimate of an *odds ratio*, it means that we are 95-percent certain that the true population odds ratio (which is unknown) lies within the confidence interval. With respect to the intervals surrounding an odds ratio, the association is positive and statistically significant if the interval values are both positive and the interval does not encompass the value of 1.0 (i.e., the value of no association). Conversely, if the interval values are both negative, the association is negative and statistically significant.
Odds ratio: A measure of association between two variables (e.g., an alcohol use disorder and a drug use disorder).
Simple random sample: A method of drawing samples such that each person, element, or unit has an equal probability of being selected.
Stratification: The classification of all persons, units, or elements of interest into comprehensive, mutually exclusive categories.
Variance estimation procedures: A technique that allows estimation of the amount of dispersion around a measure of data, such as a percentage or mean.
